# Seasonal Variations in the Composition and Physicochemical Characteristics of Sheep and Goat Milks

**DOI:** 10.3390/foods11121737

**Published:** 2022-06-14

**Authors:** Siqi Li, Munkhzul Delger, Anant Dave, Harjinder Singh, Aiqian Ye

**Affiliations:** Riddet Institute, Massey University, Private Bag 11 222, Palmerston North 4442, New Zealand; munkhzuldelger@gmail.com (M.D.); a.dave@massey.ac.nz (A.D.); h.singh@massey.ac.nz (H.S.)

**Keywords:** sheep milk, goat milk, seasonal variation, composition, calcium, fatty acids, heat treatment, heat stability

## Abstract

There has been growing consumer interest in sheep and goat milk products as alternatives to cow milk products. The physicochemical characteristics of milk vary not only between ruminant species, but also during different seasons; they determine the nutritional quality and processing properties of the milk. In this study, we characterized sheep and goat milks from New Zealand over the seasons for their composition (macronutrients, macro- and micro-minerals, fatty acids, and proteins) and physicochemical properties (e.g., ionic calcium, fat globule size, casein micelle size, viscosity, and melting behavior of milk fat). Heat-induced (95 °C for 5 min) protein interactions and changes in the physical properties of the milks were also investigated. The compositional and structural features of sheep and goat milks were identified and compared with those reported for cow milk. Seasonal variations in the milk characteristics were more pronounced for sheep milk than goat milk and were probably affected by the production systems. Sheep milk, particularly in the late season, had the largest heat-induced increases in casein micelle size and viscosity, probably arising from the greater casein–whey protein and casein–casein interactions during heat treatment. This study provides comprehensive information on the properties of sheep and goat milks and highlights the interaction effects between species, season, and processing.

## 1. Introduction

Sheep and goat milks have important roles in nutrition and food culture in many parts of the world. Worldwide, the production of sheep and goat milks has seen rapid growth in the past decades, and is projected to increase further by 26 and 53%, respectively, by 2030 [1]. The growing consumer demand for sheep and goat milks arises from their perceived health benefits and the increasing connoisseur interest in their products. The sheep and goat milk industries in New Zealand have seen promising growth in the export market in recent years, with great potential for future development. 

The composition and physicochemical properties of milk vary naturally between ruminant species [2,3,4]. Well-known characteristics of sheep and goat milks that are different from those of cow milk include their higher proportion of medium-chain fatty acids (FAs), the higher contents of protein and fat in sheep milk, and the lower α_s1_-casein content of goat milk [2]. Within each species, the characteristics of the milk are affected by the stage of lactation, parity, diet, and climate, which can vary during different seasons of the year, depending on the production systems [5,6,7,8]. Seasonal variations in the composition of sheep and goat milks have been reported in Italy [9], Spain [10,11], Austria [7], and the US [12]. In New Zealand, the typical milk production system is seasonal, with the animals giving birth and starting to produce milk at the same time of the year (commonly late winter to spring) to better utilize fresh pasture as the main feed source [6,13]. In such systems, the progressing stages of lactation synchronize with the different times of the year and play an important role in affecting the composition and the properties of seasonal milk. In addition, the climate and the diet of the animal can vary in different seasons and can contribute to different milk characteristics. Previous studies have demonstrated that seasonality plays an important role in affecting the composition of cow milk in seasonal-calving countries, such as New Zealand [6,13,14]. However, the characteristics of New Zealand sheep and goat milks and the impact of seasonality have not been studied comprehensively.

The different compositions and physicochemical properties of ruminant milks influence their processing properties and processing-induced structural changes, which can further affect product properties and their digestion behaviors. For example, sheep milk and goat milk are known to have lower heat stability than cow milk [4,15,16]. Goat milk produces weaker yogurt and rennet-induced curds than cow and sheep milks [2,17,18]. Recent studies showed that the gastric coagulation behaviors of differently processed sheep, goat, and cow milks had similarities and differences, in which the interaction effect between ruminant species and processing treatments played a role [19] and influenced the gastric digestion and emptying of the milk components [20,21].

This study aims to characterize the composition and physicochemical properties of sheep and goat milks from New Zealand comprehensively and to understand the impacts of seasonal variations and heat-induced changes on the characteristics of the milks. The results are discussed in comparison with those reported for cow milk to highlight the differences between the species and the interaction effects between species, seasonality, and processing. The results lay the foundation for a better understanding of the nutritional quality, processing properties, and digestive dynamics of sheep and goat milks.

## 2. Materials and Methods

### 2.1. Milk Sampling

The sheep and goat milks were sampled in Waikato, the main region of sheep and goat milk production in New Zealand. On collection days, fresh sheep milk and goat milk delivered to FoodWaikato (Hamilton, New Zealand) were sampled. The sheep milk was collected from two suppliers, Spring Sheep Milk Co. and Maui Milk Co., Ltd. (Hamilton, New Zealand). Raw milk from both suppliers was mixed in a 1:1 (*wt*/*wt*) ratio and used for further processing and analysis. The sheep milk was produced by traditional spring-lambing herds and was sampled throughout the 2019–2020 season (August 2019 to February 2020). The milking seasons of the sheep milk were divided as early season, mid-season, and late season, defined as 20–60, 60–130, and 130–180 days in milk, respectively. The goat milk was provided by Cilantro Cheese Ltd. (Hamilton, New Zealand). It was produced in a year-round system by mixing spring-kidding herds and autumn-kidding herds; this represents the common dairy goat management practice in the Waikato region. The goat milk was sampled in three seasons: spring, summer, and winter from September 2019 (spring) to July 2020 (winter). The feeding regimes of the animals were managed by the respective suppliers.

Three different batches of milk were collected and analyzed for each species in each season. Part of the raw milk was skimmed at 3000× *g* for 15 min for further analyses. Milk serum was separated from the skim milk by acid precipitation for analyzing native whey proteins [6] and by ultracentrifugation (63,000× *g* for 60 min at 20 °C) for analyzing soluble minerals and proteins. Sodium azide (0.02% *wt/wt*) was used to preserve the milk samples. Aliquots of milk were stored at –80 °C before compositional analyses.

### 2.2. Macronutrient and Mineral Analysis

The proximate compositions of the milks (fat, protein, lactose, and total solids) were measured using a MilkoScan FT1 (Foss Electric, Hillerød, Denmark). Milk minerals were analyzed at Hill Laboratories (Hamilton, New Zealand). Calcium (Ca), magnesium (Mg), potassium (K), sodium (Na), and phosphorus (P) were determined by inductively coupled plasma–optical emission spectrometry. The concentrations of copper (Cu), iodine (I), selenium (Se), and zinc (Zn) were measured by inductively coupled plasma–mass spectrometry. Before the inductively coupled plasma analysis, samples for the determination of I and Se were extracted using tetramethylammonium hydroxide [22], whereas samples used to analyze Ca, Mg, K, Na, P, Cu, and Zn were digested with nitric acid and hydrochloric acid (method 3030F; [23]). The chloride (Cl) content was determined by potentiometric titration (AOAC 971.27). The soluble fractions of Ca, Mg, and P were determined in skim milk serum separated from the raw skim milk by ultracentrifugation followed by filtering through Amicon^®^ Ultra-15 filters (10 kDa). The ionic Ca concentration was determined as described in Li et al. [6] using a calcium-selective electrode (Orion 9720BNWP; Thermo Fisher Scientific, Waltham, MA, USA).

### 2.3. Fatty Acid Composition and Melting Profile of Milk Fat

Milk fat was extracted using hexane and isopropanol and washed using a Na_2_SO_4_ solution as described by Hara and Radin [24]. The final fat extract in hexane was evaporated to dryness using a stream of nitrogen. 

The extracted milk fat was methylated using sodium methoxide, as described previously [25]. The methylated samples were analyzed for FA composition on a gas chromatograph (Shimadzu Nexis GC-2030) equipped with a flame ionization detector and a Restek Rx 2330 column (105 m × 0.25 mm ID, 0.20 µm film thickness). The injector temperature and the detector temperature were set at 260 and 265 °C, respectively. The injection volume was 1 µL and the split ratio was 50:1. The oven temperature was initiated at 75 °C and held for 5 min, increased to 175 °C at a rate of 15 °C/min and held for 27 min, then increased to 250 °C at a rate of 15 °C/min, and finally held for 13 min. The *cis*-9,*trans*-11 isomer of conjugated linoleic acid (CLA) in the milk fat was identified and is referred to as CLA in this study. The ratios of product and substrate FAs of stearoyl-CoA desaturase (SCD), namely C10:1/C10:0, C14:1/C14:0, C16:1/C16:0, C18:1 c9/C18:0, and CLA/C18:1 t11, were reported as indicators for SCD activity.

The melting behavior of the extracted milk fat was analyzed using differential scanning calorimetry on a DSC Q 2000 (TA Instruments, New Castle, DE, USA), as described in Li et al. [14]. Based on the melting thermogram, the estimated proportions of milk fat melt in different temperature ranges were calculated and defined as the low-melting fraction (LMF, <5 °C), the medium-melting fraction (MMF, 5–20 °C), and the high-melting fraction (HMF, >20 °C).

### 2.4. Protein Composition 

The protein compositions of the milks and milk serums were determined using high-performance liquid chromatography (HPLC). Samples were prepared for HPLC as described in Bobe et al. [26]. HPLC was performed on an Aeris Widepore 3.6 µm XB-C18 RP column (Phenomenex, Torrance, CA, USA) using a method modified from previous studies [26,27]. The mobile phase consisted of a solvent A (water:acetonitrile:trifluoroacetic acid = 900:100:1) and a solvent B (water:acetonitrile:trifluoroacetic acid = 100:900:1). The separation gradient was started at 27% solvent B, was increased to 32% in 2 min, then to 45.6% in 29 min, and to 50.2% in 1 min, was held for 2 min, was returned to the initial condition in the next 2 min, and was held for another 9 min. The total run time was 45 min and the flow rate was set at 0.6 mL/min. The proteins were detected at a UV wavelength of 220 nm. The protein composition was indicated by the percentages of peak areas of individual proteins in the total peak area of all proteins in the HPLC chromatogram. The proportions of serum-phase κ-casein and β-casein were also analyzed by calculating the protein peak area in milk serum (separated by ultracentrifugation as described in Section 2.1) as a percentage of that in the corresponding skim milk.

### 2.5. Physicochemical Properties and Heat-Induced Changes

The pH, ethanol stability, and fat globule size of the raw sheep and goat milks were analyzed as described by Li et al. [6]. Heat-induced changes in casein micelle size, viscosity, and whey protein–casein micelle interactions were determined in the seasonal sheep and goat milks heated at 95 °C for 5 min. This heat treatment was chosen as it induces significant protein interactions and because it is representative of yogurt milk-processing conditions [6]. The extents of whey protein denaturation and the distribution of denatured whey proteins between the micellar phase and serum phase in heated milk were determined by HPLC analysis of whey proteins in milk serums separated by acid precipitation and ultracentrifugation, and presented as percentages in the total whey proteins in skim milk, as described in Li et al. [6]. Native whey proteins were determined as those remained in the supernatant following acetic acid precipitation to pH 4.6. Soluble whey proteins were defined as those present in the serum following ultracentrifugation at 63,000× *g* for 60 min. Soluble aggregates of denatured whey proteins were calculated as the difference between soluble and native whey proteins. Micelle-bound whey proteins were calculated as the difference between total whey proteins in the skim milk and the soluble whey proteins in the ultracentrifugation serum. The casein micelle size of the raw and heated skim milks was analyzed by a Malvern Zetasizer Nano ZS (Malvern Instruments, Malvern, UK) as described previously [19]. The viscosity of the whole milk (raw and homogenized-heated at 20/5 MPa; 95 °C for 5 min) was measured on a rheometer (AR-G2; TA Instruments, Cheshire, UK) paired with concentric cylinder geometries. The sample volume was 20 mL and the measurement temperature was 20 °C. A shear rate sweep from 0.01 to 1000 s^–1^ was performed over 3 min. The measured viscosity stabilized in the shear rate range of 10–100 s^–1^, in which the mean viscosity was recorded. 

### 2.6. Statistical Analysis

Minitab 19 was used for statistical analysis. Significant differences between species and seasons were analyzed using independent sample t-tests and one-way analysis of variance (ANOVA) followed by Tukey’s post hoc test. A correlation analysis was performed to determine significant correlations and Pearson correlation coefficients (*r*) between parameters. Standard deviations were used to indicate the variation of the means and are plotted as the error bars in the figures. A coefficient of variation (CV) was used as an indicator for the magnitude of the variation. 

## 3. Results and Discussion

### 3.1. Compositional and Physicochemical Properties of Sheep and Goat Milks

#### 3.1.1. Composition of Macronutrients and Minerals

The compositions of macronutrients and minerals in the goat and sheep milks are presented in Table 1. Consistent with previous reports, the sheep milk contained significantly higher protein, fat, lactose, and total solids than the goat milk (*p* < 0.001 in all cases). The macrominerals Ca, Mg, and P were also higher in the sheep than in the goat milk, presumably because a considerable proportion of these minerals are associated with the casein micelles [28]. The concentrations of Ca, Mg, and P were significantly correlated with the protein contents of the milks (*p* < 0.001). K and Cl were significantly higher in the goat than in the sheep milk (*p* < 0.001), consistent with the report by Mayer and Fiechter [7]. No difference was found in the concentrations of Na between the two species. Among the microminerals, Cu, Se, and Zn were significantly higher in the sheep milk (*p* < 0.05). The iodine content did not differ between the goat and sheep milks. The mineral composition results of the goat and sheep milks were largely consistent with those reported previously [2,29], except that both the goat and sheep milks in the present study contained less Cu and more Se. The goat milk contained significantly higher percentages of soluble Ca, Mg, and P (29.7, 69.2, and 49.7%, respectively) than the sheep milk (19.6, 58.2, and 38.7%), in agreement with the study of De La Fuente et al. [30]. Moreover, the ionic Ca concentration was higher in the goat (3.18 mM) than the sheep milk (2.70 mM), despite the total Ca in the goat milk being on average 43% lower than in the sheep milk. On average, ionic Ca made up 10.8% of the Ca in the goat milk, more than double that in the sheep milk (5.2%). In cow milk, soluble Ca, Mg, and P make up approximately 30, 65, and 54% of the total, respectively [28], close to the values of the goat milk in the present study. Ionic Ca makes up approximately 7–9% of the total Ca in cow milk [6,31], in between those of the goat and sheep milks in the present study.

With respect to the impact of seasonality, for the sheep milk, the contents of protein and fat increased and the lactose content decreased in the late season. This was consistent with the changes driven by the progressing stage of lactation reported previously in both sheep milk [7,9,10] and cow milk [6,13]. The Ca content of the sheep milk did not vary significantly over the seasons, largely consistent with the report by Sevi et al. [9]. The ionic Ca concentration had an insignificant increasing trend over the seasons (*p* > 0.1). The P content was significantly lower in the mid-season sheep milk, although the range of variation was fairly small. The late-season sheep milk contained the highest amounts of Mg, Na, and Cl, and the lowest amount of K (Table 1). The increases in Na and Cl and the decrease in K in late lactation were consistent with the lactational trends of cow milk [28]. The soluble fractions of Ca, P, and Mg did not vary significantly. For the microminerals, the early season sheep milk contained the highest concentrations of Cu and Zn, whereas Se was highest in the mid-season. 

For the goat milk, the contents of fat, protein, and total solids were lowest in summer (*p* < 0.05). However, the extent of variation for the protein concentration in the goat milk was quite small (CV 2.4%), similar to that of lactose (CV 1.2%). As for minerals, Ca and P were highest in the winter goat milk, whereas no significant difference was found for the proportions of soluble minerals (Ca, P, and Mg). The summer goat milk contained the highest concentrations of K, Cl, and I and the lowest concentrations of Cu and Zn. The observed seasonal differences in fat, protein, Ca, and P were largely consistent with those reported by Mayer and Fiechter [7], in an asynchronous-kidding goat herd. Similarly, Kljajevic et al. [32] also reported that the fat content in goat milk had the greatest variation over the seasons; it was lowest in the summer months. It was correlated negatively with the solar radiation duration and the temperature humidity index [32]. Cow milk produced in nonseasonal calving counties also contained the lowest fat in summer, which has been associated with a higher proportion of fresh grass in the diet [5,33].

#### 3.1.2. Fatty Acid Composition and Melting Properties of Milk Fat

Table 2 presents the FA compositions and the different melting fractions of the sheep and goat milks. As with previous studies, the most abundant FAs in both the sheep milk and the goat milk were C10:0, C14:0, C16:0, C18:0, and C18:1 c9 [2,7]. Among the individual FAs, the goat milk fat was richer in C18:0, C18:1 c9, C18:2, C20:0, and C20:4 than the sheep milk fat. Driven by the difference in C18:1 c9 between the goat milk (21.6 ± 2.2%) and the sheep milk (15.0 ± 1.1%), the goat milk also contained significantly higher total monounsaturated fatty acids (MUFA) than the sheep milk. No significant difference between the two species was found for C8:0, C10:0, C16:0, iso C17, and anteiso C17. Most other individual FAs were significantly higher in the sheep milk than the goat milk, including C4:0, C6:0, and C12–C15 FAs, and the minor C18:1 FAs (except for C18:1 c9), C18:3, and CLA. The desaturation ratios of C10, C14, and C16 were significantly higher in the sheep milk than the goat milk, indicating a greater activity of SCD in sheep than in goats, which agreed with that reported by Tsiplakou et al. [34]. The proportions of milk fat in the different melting temperature ranges were generally similar for the goat and sheep milk fats. The LMF was 3.3% higher in the sheep milk fat than in the goat milk fat (*p* < 0.05), whereas the differences in MMF and HMF were nonsignificant. 

The FA composition results were compared with those reported previously [2,7,35,36,37,38,39,40]. The concentrations of C4:0, C15 FAs, and C17 FAs were rather consistent among different studies. The results in this study were in line with most previously reported data; C4 and C15 FAs were higher in the sheep milk fat. The reported proportions of C6:0 and C8:0 in previous studies fell in a fairly small range, but there was no consistent trend to indicate whether sheep milk fat or goat milk fat contained more of either FA. C10:0, C12:0, C14:0, and C16:0 make up 40–50% of the FAs in goat and sheep milks. In this study, all these FAs were found in the lower range in the goat milk, whereas the sheep milk contained higher C10–C14 FAs than what was found in previous studies. For both species, the contents of C18:0 and C18:1 c9 in milk fat were reported in a very wide range because of marked dietary influences [40]. In this study, these two FAs in the sheep milk were lower than average in the reported range. The CLA contents in the milk fat in the present study were fairly high, particularly for the sheep milk. The higher prevalence of pasture feeding in New Zealand may have contributed to the overall lower saturated FAs and the higher CLA in the goat and sheep milks [40].

Compared with cow milk produced in New Zealand [13,14], the saturated C6–C12 FAs were higher in both the sheep and goat milks, whereas the contents of C16:0 were lower in the sheep and goat milks than in cow milk. Probably arising from these differences in FA composition, the LMF of the goat and sheep milk fats (55–60%) was greater than that of cow milk fat (~30%); greater proportions of both the MMF (~40%) and the HMF (~30%) were reported in cow milk fat than in the sheep and goat milk fats [14,41].

Overall, the late-season sheep milk fat had the most distinctive FA composition, including the lowest C4:0–C10:0 saturated FAs and the highest concentrations of C14:0, C16:0, and C20:0. In addition, all desaturation ratios were the highest in the late-season sheep milk (*p* < 0.05), indicating a pronounced impact of the stage of lactation on the SCD activity. The desaturation products C10:1, C14:1, and C16:1 were also the highest in the late season. All C18 FAs tended to be higher in the early season, although statistically only C18:1 t11, C18:2, and C18:3 were significantly higher in the early season sheep milk fat than in either the mid- or late-season sheep milk fat. The polyunsaturated fatty acids in the sheep milk fat were significantly higher in the early season. The seasonal variations in FA composition were largely consistent with those reported over the lactation by Casoli et al. [42]. They also found pronounced decreasing trends in C4:0–C10:0 and C18:2 and increasing trends in C16:1 and C20:0 as the lactation progressed. Higher SCD activity in late lactation has been reported in sheep milk [11] and cow milk [14,43,44]. With respect to the melting fractions of the sheep milk fat, the proportion of HMF was the highest in the late season. It was correlated positively with the content of C16:0 (*r* = 0.944, *p* < 0.001) and negatively with C4–C10 saturated FAs (*p* < 0.05 in all cases).

The seasonal variations in the FA composition of the goat milk appeared to be less systematic than those of the sheep milk. In the spring, the goat milk fat contained the highest proportions of C6:0, C18:1 t10, and C18:2 and the lowest proportions of C17 FAs and C20:0. The summer goat milk fat had the lowest C4:0 and C10:0 and the highest C14, C14:1, and C15 FAs and iso C16. The winter milk contained the highest C16:1, many of the C18 FAs (C18:0, C18:1 t9, C18:1 t11, C18:1 c9, and CLA), C20:0, and the total MUFA. Siefarth and Buettner studied the FA composition of goat milk from two farms in Germany in summer and winter [45]. Consistent with the present study, they reported that winter goat milk contained higher C18:0 and total MUFA and lower C14:0 than summer goat milk. However, the overall difference in the FA compositions of the winter and summer goat milks was rather small and some of the significant seasonal differences were significant in only one of the two farms studied [45]. Unlike the sheep milk, the SCD activity of the goat milk, as indicated by ratios between desaturation products and substrates, did not vary consistently over the seasons (Table 2). The C14 desaturation ratio did not vary significantly with the season; it is regarded as the best indicator of SCD activity because desaturation is the sole source of C14:1 in milk fat [46]. In the goat milk fat, the LMF increased and the MMF decreased significantly in the winter (*p* < 0.05). Higher C18 FAs and lower C8–C14 FAs were significantly correlated with a higher LMF and a lower MMF (*p* < 0.05).

#### 3.1.3. Protein Composition

**Figure 1:**
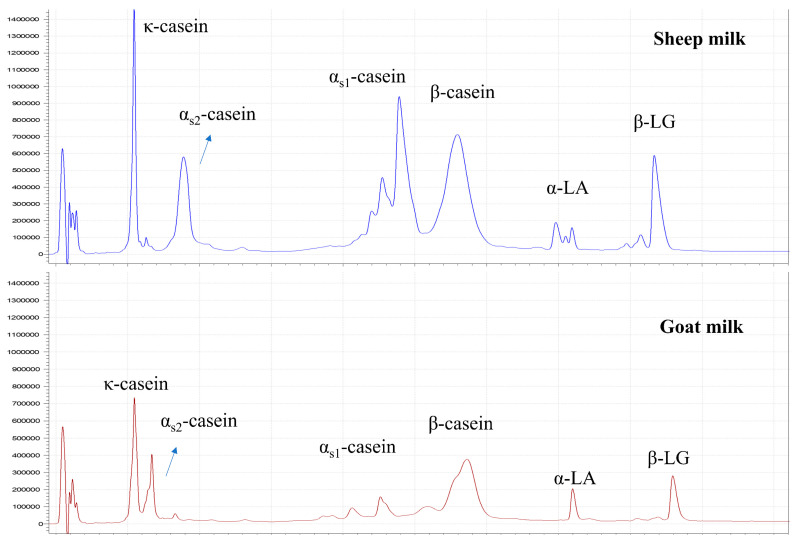
High-performance liquid chromatography profiles of proteins in sheep milk and goat milk.

Representative HPLC profiles of proteins in sheep milk and goat milk are shown in Figure 1. Based on HPLC results, the protein compositions of the goat and sheep milks were analyzed and are presented in Table 3. The most abundant protein in the goat milk was β-casein, consistent with previous reports [2,3]. The different genetic polymorphisms of α_s1_-casein are well known to greatly influence its concentration in goat milk, ranging from 0 to 3.5 g/L milk [2,47]. α_s1_-Casein was at a low level in the goat milk in the present study, making up about 11.0% of the total caseins. The sheep milk was most abundant in α_s1_-casein and β-casein (Table 3). Previous studies reported varying proportions of α_s1_-casein and β-casein in sheep milk [36,48,49,50,51], which, similar to goat milk, are also influenced by protein genotypes [50,51]. Considerable proportions of κ-casein (22.5%) and β-casein (16.0%) were present in the serum phase of the goat milk, and were higher than those in the sheep milk (Table 3) and cow milk (7 and 4%, respectively, revisiting data from Li et al. [6]). Among the whey proteins, α-lactalbumin (α-LA) made up a larger proportion in the goat milk than in the sheep milk (Table 3). This was consistent with the finding of Moatsou et al. that the β-lactoglobulin (β-LG):α-LA ratio of goat milk was lower than that of cow and sheep milks [52].

Significant seasonal variations in the proportion of individual proteins were found for ĸ-casein and β-LG in both sheep and goat milk (Table 3). Winter goat milk was the highest in β-LG (12.0%) and the lowest in ĸ-casein (15.9%). For sheep milk, the proportion of ĸ-casein was lowest in the early season (10.1%), whereas β-LG was the highest in the late season (9.7%). Yet, the extents of seasonal variation of these proteins for both sheep and goat milks were rather small (CV ranged from 4.0 to 8.6%). Besides, the proportion of serum κ-casein in total κ-casein in goat milk was the highest in spring (28.3%); the proportion of serum β-casein in total sheep milk β-casein was the highest in the early season (9.4%).

#### 3.1.4. Physicochemical Properties

Table 4 shows some physicochemical characteristics of the sheep and goat milks. The sheep milk had a larger fat globule size, a smaller casein micelle size, and a higher viscosity than the goat milk (*p* < 0.05), largely consistent with previous results [2,3]. The ethanol stabilities of the goat and sheep milks were similar, around 50%. It is well known that the colloidal stability of the casein micelles in goat and sheep milks is lower than in cow milk, which typically has an ethanol stability greater than 70–75% [4,53]. 

Seasonality significantly affected all the determined physicochemical properties of the sheep milk. Late-season sheep milk had the lowest pH, lowest ethanol stability, and highest viscosity (Table 4). The highest protein and total solids concentrations of the late-season sheep milk (Table 1) probably contributed to its lower ethanol stability and higher viscosity. The casein micelle size was the largest in the early season and the smallest in the mid-season. It had a strong negative correlation with the proportion of κ-casein in the total protein in the sheep milk (*r* = –0.959, *p* < 0.001). As κ-caseins are mostly located on the surfaces of the casein micelles, it has been suggested that a higher proportion of κ-casein in milk protein is associated with a smaller casein micelle size [54]. However, this correlation was not found to be pronounced in milk produced by individual cows [55,56]. For the goat milk, seasonal variation was significant only for ethanol stability, which was the highest in spring. No other characteristics of the goat milk varied significantly over the seasons (Table 4).

### 3.2. Behaviour of Proteins and Physical Changes in Milk upon Heat Treatment

#### 3.2.1. Whey Protein Denaturation and Association with the Casein Micelles

**Figure 2:**
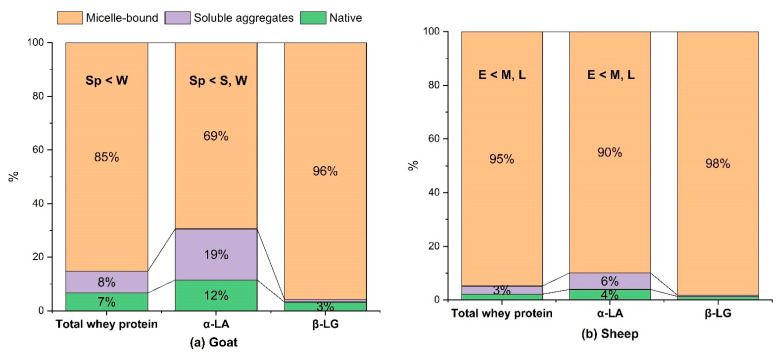
Distribution of whey proteins in the (**a**) goat milk and (**b**) sheep milk heated at 95 °C for 5 min (fractions below 3% are not labeled); significant seasonal differences in the micelle-bound fraction are indicated (*p* < 0.05); Sp, spring; S, summer; W, winter; E, early season; M, mid-season; L, late season.

The proportions of whey proteins in the heated (95 °C for 5 min) goat and sheep milks that were associated with the casein micelles, formed soluble aggregates in the serum phase, or remained native are presented in Figure 2. The heat treatment of 95 °C for 5 min induced high levels of whey protein denaturation in both the sheep milk and goat milk (>90% in all samples). However, there was a small but significant difference in the extent of whey protein denaturation between the heated sheep milk (97.8 ± 0.8%) and the heated goat milk (93.4 ± 2.0%, *p* < 0.001). This difference was mainly driven by the different extents of denaturation of α-LA, considering that β-LG was highly denatured in both the goat milk and sheep milk (96.7 and 98.7%, respectively). α-LA was denatured relatively more slowly in the goat milk (88.5%) than in the sheep milk (96.1%) after being heated at 95 °C for 5 min. The majority of the denatured whey proteins were associated with the casein micelles in both the sheep milk and goat milk. The heated sheep milk contained a higher proportion of micelle-bound whey proteins among the denatured whey proteins than the heated goat milk (*p* < 0.001). Similar to the trend observed for whey protein denaturation, α-LA displayed different tendencies for micelle association between the two species, whereas denatured β-LG was almost completely micelle-bound in both types of milk (~99.0%). The percentage of denatured α-LA that remained in the serum was 21.5% in the goat milk and 6.4% in the sheep milk.

The differences in the extent of whey protein denaturation and micelle association under the heating conditions used in this study may be attributed to the higher β-LG:α-LA ratio in the sheep milk than in the goat milk (Table 3), given that β-LG is more prone to heat-induced denaturation than α-LA and can promote the denaturation of α-LA via disulfide bonding [6,57]. In addition, the higher protein concentration of the sheep milk may also promote protein–protein interactions, including those between α-LA and denatured β-LG and between denatured whey proteins and the casein micelles. The different levels of α-LA forming soluble aggregates in the sheep and goat milks, despite the exclusive association of denatured β-LG, suggests that denatured α-LA in goat milk may form complexes with other caseins in the serum phase with less involvement of β-LG. Pesic et al. reported that the micelle-bound ĸ-casein–whey protein complexes in goat milk also involved α_s_-casein and β-casein, whereas only ĸ-casein formed complexes with the whey proteins in heated cow milk [58]. It is unclear whether the serum-phase caseins found in higher proportions in the goat milk formed complexes with α-LA and contributed to the higher proportion of serum-phase α-LA in the heated goat milk than in the heated sheep milk.

Previous studies reported that cow milk heated under comparable conditions had lower levels of whey protein–casein micelle association and a greater proportion of denatured whey proteins in the serum phase than both the sheep milk and the goat milk in the present study [6,58,59]. The proportion of whey proteins associated with the casein micelles in cow milk was reported to reach a plateau of 70–80% during prolonged heating (up to 60 min) at temperatures up to 100 °C [59]. In contrast, in sheep milk, most whey proteins were found to be associated with the casein micelles after heating at 80–90 °C for 30 min [16]. Similarly, Pesic et al. reported that, in goat milk heated at 90 °C for 10 min, denatured whey proteins were exclusively associated with the casein micelles, whereas, in cow milk undergoing the same heat treatment, about 30% of the denatured whey proteins formed soluble complexes [58].

#### 3.2.2. Casein Micelle Size and Milk Viscosity

**Figure 3:**
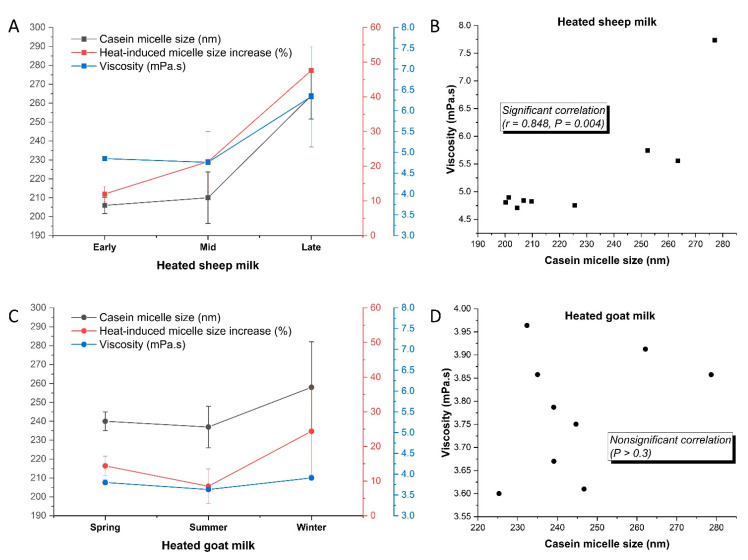
Casein micelle size (black), heat-induced micelle size increase (red), and viscosity (blue) of homogenized-heated (95 °C for 5 min) (**A**) sheep milk and (**C**) goat milk (Y-axes are indicated by corresponding colors in the legends) and correlations between casein micelle size and viscosity of heated (**B**) sheep milk and (**D**) goat milk.

The heat treatment (95 °C for 5 min) significantly increased the mean casein micelle diameter of the sheep and goat milks by 48 and 33 nm, respectively, corresponding to increases of 27 and 16% (Figure 3A,C). In contrast, heating cow milk under similar conditions (90 °C for 6 min) increased the casein micelle diameter by only approximately 10 nm or 6% [6]. Similarly, Raynal and Remeuf reported that the casein micelle size in cow milk remain unchanged when heated at 90 °C for up to 10 min, whereas heated goat and sheep milks had 25 and 75% increases, respectively, in micelle size [57].

The mechanisms of heat-induced modification of the micelle size in the different ruminant milks probably differ because of their different physicochemical characteristics. It has been well demonstrated in cow milk that the association of denatured whey proteins with the casein micelles is the main contributor to the minor increase in micelle size upon heating at temperatures under 100 °C, although some aggregation of the casein micelles may also play a part [59]. Li et al. reported that heating cow milk at the UHT condition of 140 °C for 5 s induced a much greater increase in the mean casein micelle size than heating at 90 °C for 6 min, despite the lower whey protein–casein micelle association level in the UHT milk [6]. It was thus suggested that the minor aggregation between the casein micelles in UHT milk played an important role in increasing the measured micelle size [6]. Goat and sheep milks are known to be less heat stable than cow milk and are more prone to fouling during heat treatment [4]. As a result, the coagulation between the casein micelles when heated at below 100 °C may occur to greater extents in goat and sheep milks and contributes more to the heat-induced increase in casein micelle size than in cow milk. For cow milk, because of its greater heat stability, only more intense heating conditions (such as UHT treatment) can result in noticeable aggregations between the casein micelles that lead to substantial increases in casein micelle size and sediment formation [6,27,60]. Raynal and Remeuf suggested that the lower colloidal stability in goat and sheep milks could favor the aggregation of micelles and increase the proportion of larger micelles following heat treatment [57]. Therefore, the greater increase in the casein micelle size in the goat and sheep milks upon heating at around 90 °C may have also been caused by the aggregation of micelles in addition to the association with the whey proteins. This was in agreement with a recent study in which the mean casein micelle size in heated sheep skim milk increased under more intense heating conditions despite the whey protein–casein micelle association level plateauing at around 95%, which was attributed to the aggregation between the casein micelles [16]. Using transmission electron microscopy, the authors also observed aggregated casein micelles in sheep milk heated at 85 and 90 °C for 30 min [16], which was found only in UHT cow milk at a much lower extent of aggregation (140 °C for 5 s, Li et al. [27]). Similarly, Yuan et al. recently reported that the casein micelles in goat milk micellar casein concentrate were more susceptible to aggregation than those in their cow milk counterparts, as evidenced by a greater increase in particle size and the greater extent of aggregation observed by transmission electron microscopy [61]. Possible reasons for the greater heat-induced increases in casein micelle size in sheep and goat milks may include: the higher protein concentration of sheep milk, which promotes protein–protein interactions [57]; the higher concentrations of ionic Ca, particularly for goat milk (Table 1), which promotes both the association between whey proteins and casein micelles and the micelle–micelle aggregation [6,62]; and the lower hydration and higher mineralization of sheep and goat milk casein micelles [4].

#### 3.2.3. Seasonal Variations in Heat-Induced Changes

Early season sheep milk had the lowest extents of whey protein denaturation and micelle association (Figure 2b). The extent of whey protein denaturation was correlated with the proportion of whey proteins (α-LA and β-LG) in all proteins and the ionic Ca concentration in the sheep milk (*p* < 0.05). Similarly, in heated cow milk (90 °C for 6 min), Li et al. found that the heat-induced denaturation of the whey proteins and their association with the casein micelles was significantly correlated with the ionic Ca concentration [6]. It was suggested that ionic Ca can bind to the negatively charged proteins and facilitate their associations. The late-season sheep milk had the greatest heat-induced increase in casein micelle size at 47.6 ± 6.5%, higher than that in earlier seasons (around 20%, Figure 3A). The pronounced seasonal variation in the heat-induced increase in casein micelle size in the sheep milk was correlated with the concentrations of protein and ionic Ca (*r* = 0.87 and 0.82, respectively, *p* < 0.01 in both cases). They can promote whey protein–casein micelle association and micelle–micelle aggregation, both of which could lead to an increased average casein micelle size. The heat-induced micelle–micelle aggregation, as discussed above, was probably the more important contributor to the variation in the measured casein micelle size in the present study. Similar to the seasonal effects found in the sheep milk in the present study, Li et al. reported that cow milk that underwent UHT treatment (140 °C for 5 s) had the largest casein micelle size in the late season in New Zealand, which was also correlated significantly with the protein content and the ionic Ca concentration of the milk [6]. The mean viscosity of the heated sheep milk was higher in the late season (6.34 mPa·s) than in the early and mid-seasons (around 4.80 mPa·s, Figure 3A), although the difference was just below the level of significance (*p* = 0.055). The large variation in the viscosity of the heated late-season sheep milk, which could reduce the significance of seasonal differences, arose mainly from the last sample collected during the season, which had the highest viscosity (7.73 mPa·s) and the largest casein micelle size (277 nm) of all samples (Figure 3B). The viscosity of the heated sheep milk had significant correlations with its protein content, ionic Ca concentration, and casein micelle size (*p* < 0.05, Figure 3B), but not with the extent of whey protein–casein micelle association (*p* > 0.1). Previous studies have reported that the heat-induced association of whey proteins with the casein micelles increases the volume fraction of the micelles, resulting in a higher viscosity of the milk [59,63]. In the present study, the high level of whey protein–casein micelle association in the heated sheep milk (92–97%) was the probable reason for its nonsignificant correlation with the viscosity. This suggested that the increased attraction between the casein micelles and their minor aggregation during heating was probably the main contributor to the increased viscosity. The aggregation of particles in a dispersion increases viscosity by immobilizing the surrounding liquid, increasing the effective volume fraction, and reducing the maximum packing fraction of the particles [64]. The late-season sheep milk, with its highest protein content and ionic Ca concentration, was likely to favor micelle–micelle aggregation the most over the seasons, as indicated by the most pronounced increase in the average casein micelle size (Figure 3A). In addition, the late-season sheep milk also had the highest concentration, and thus the highest volume fraction, of total milk solids (Table 1), further amplifying the effect of greater extents of micelle–micelle aggregation on increasing the viscosity of the heated milk.

For the goat milk, the extents of whey protein denaturation and whey protein–casein micelle association were the highest in winter and the lowest in spring, which was driven by the difference in α-LA because β-LG was almost exclusively associated with the casein micelles in the heated goat milk (Figure 2a). The higher proportion of β-LG in the winter goat milk probably played a part in promoting whey protein denaturation, as indicated by their significant correlation (*r* = 0.786, *p* = 0.012). Similar correlations have been reported in seasonal cow milk in New Zealand; this was ascribed to the role of β-LG in forming disulfide bonds and catalyzing the denaturation of α-LA [6]. Seasonality did not affect the heat-induced increase in casein micelle size, whereas the viscosity of the heated (95 °C for 5 min) goat milk was the lowest in the summer (Figure 3C), which was correlated with the contents of fat and total solids in the goat milk (*p* < 0.01). Unlike the sheep milk (Figure 3B), there was no correlation between the casein micelle size and the viscosity of the heated goat milk (Figure 3D). Probably, the viscosity of the goat milk was not sensitive to the heat-induced change in micelle size and the potential micelle–micelle aggregation because of its lower volume fraction of total solids (Table 1).

## 4. Conclusions

This study demonstrated the compositional and physicochemical characteristics of sheep and goat milks from New Zealand. The effects of seasonal variations and the overall differences between sheep, goat, and cow milks were also discussed. In addition to the well-known differences in the compositions of macronutrients, the differences in the mineral and protein fractions of sheep and goat milks, including the different proportions of soluble/nonmicellar minerals and proteins, were demonstrated. The varying fatty acid composition indicated differences in SCD activities and affected the melting properties of the milk fat of different species and seasons. Physicochemical properties, such as casein micelle size and fat globule size, also varied considerably between the species. The seasonal variation patterns of many characteristics of the sheep milk, including macronutrient composition, mineral composition, FA composition, and fat globule size, were consistent with those reported during the lactation cycle of sheep or cows. This probably arose from the seasonal-lambing practice of the sheep herds. In contrast, goat milk produced from a nonseasonal kidding system showed seasonal variation patterns that were comparable with those reported in goats and cows from nonseasonal production systems. Processing-induced changes varied between the milks from the different species. The extents of heat-induced (95 °C for 5 min) whey protein–casein micelle association and the increases in casein micelle size were greater in the sheep milk than in the goat milk, and both were higher than those reported for cow milk under comparable conditions. Moreover, the late-season sheep milk had the highest heat-induced increase in casein micelle size, which correlated with its increased viscosity. Minor aggregations between the casein micelles probably played a key role in altering the physical properties of the heated sheep milk and, to a lesser extent, of the heated goat milk. Many of the variations in heat-induced changes can be explained by the differences (between seasons and species) in the concentrations of protein and ionic Ca, and the proportions of α-LA and β-LG in the milk proteins. This study provided comprehensive information on sheep and goat milks over the seasons, which could inform product development and further studies on the processing and functional properties of sheep and goat milks and their components.

## Figures and Tables

**Table 1:** Compositions of major components and minerals in the seasonal goat and sheep milks.

	Goat			Sheep		
	Mean ± SD	Min–Max	Seasonal Effect *	Mean ± SD	Min–Max	Seasonal Effect *
Total solids (*wt*/*wt*%)	11.90 ± 0.53 ^b^	11.22–12.41	S < Sp, W	17.46 ± 0.60 ^a^	16.91–18.55	L > E, M
Protein (*wt*/*wt*%)	3.19 ± 0.08 ^b^	3.09–3.30	S < W	5.72 ± 0.28 ^a^	5.45–6.26	L > E, M
Fat (*wt*/*wt*%)	3.79 ± 0.39 ^b^	3.30–4.27	S < Sp, W	5.99 ± 0.44 ^a^	5.43–6.70	L > E, M
Lactose (*wt*/*wt*%)	4.40 ± 0.05 ^b^	4.28–4.45	NS	4.76 ± 0.12 ^a^	4.58–4.85	L < E, M
Ca (g/100 g)	0.114 ± 0.005 ^b^	0.108–0.121	W > Sp, S	0.200 ± 0.007 ^a^	0.189–0.210	NS
Soluble Ca (% in total Ca)	29.7 ± 1.8 ^a^	27.3–33.3	NS	19.6 ± 0.8 ^b^	18.5–20.5	NS
Ionic Ca (% in total Ca)	10.8 ± 0.8 ^a^	10.0–12.6	NS	5.2 ± 0.4 ^b^	4.9–5.8	NS
Magnesium (g/100 g)	0.014 ± 0.001 ^b^	0.013–0.015	NS	0.018 ± 0.002 ^a^	0.016–0.021	L > E, M
Soluble Mg (% in total Mg)	69.2 ± 4.3 ^a^	64.8–78.8	NS	58.2 ± 1.3 ^b^	55.4–60.0	NS
Phosphorus (g/100 g)	0.100 ± 0.003 ^b^	0.095–0.104	W > S	0.160 ± 0.003 ^a^	0.156–0.165	M < E, L
Soluble P (% in total P)	49.7 ± 3.8 ^a^	45.6–57.6	NS	38.7 ± 2.1 ^b^	35.6–42.9	NS
Potassium (g/100 g)	0.203 ± 0.009 ^a^	0.195–0.220	S > Sp, W	0.132 ± 0.007 ^b^	0.119–0.138	L < E, M
Sodium (g/100 g)	0.037 ± 0.001	0.035–0.038	NS	0.042 ± 0.007	0.035–0.052	L > E, M
Chloride (g/100 g)	0.164 ± 0.008 ^a^	0.153–0.175	S > Sp, W	0.089 ± 0.012 ^b^	0.071–0.105	L > M > E
Copper (mg/kg)	0.103 ± 0.020 ^b^	0.064–0.129	S < Sp, W	0.171 ± 0.072 ^a^	0.099–0.290	E > M, L
Iodine (mg/kg)	0.251 ± 0.049	0.176–0.330	S > W	0.224 ± 0.042	0.153–0.270	NS
Selenium (mg/kg)	0.029 ± 0.004 ^b^	0.023–0.035	NS	0.036 ± 0.005 ^a^	0.030–0.044	M > E, L
Zinc (mg/kg)	3.578 ± 0.286 ^b^	3.200–4.000	S < Sp, W	5.822 ± 0.307 ^a^	5.300–6.400	E > L

^a,b^ Different superscripts indicate significant differences between goat and sheep milks (*p* < 0.05, *t* test); superscript “a” denotes a significantly higher mean, whereas “b” denotes a lower one. * Indicates significant pairwise differences between different seasons using a one-way analysis of variance (ANOVA) with Tukey’s test (*p* < 0.05). SD, standard deviation; Sp, spring; S, summer; W, winter; E, early season; M, mid-season; L, late season; Ca, calcium; Mg, magnesium; P, phosphorus; NS, nonsignificant.

**Table 2:** Fatty acid compositions (g/100 g fatty acids), fatty acid ratios, and proportions of melting fractions (%) of sheep and goat milk fats.

	Goat			Sheep		
Item	Mean ± SD	Min–Max	Seasonal Effect *	Mean ± SD	Min–Max	Seasonal Effect *
C4:0	2.62 ± 0.09 ^b^	2.45–2.74	S < Sp, W	3.44 ± 0.26 ^a^	3.00–3.78	L < E, M
C6:0	2.47 ± 0.13 ^b^	2.30–2.68	Sp > W	2.76 ± 0.26 ^a^	2.32–3.08	L < E, M
C8:0	2.73 ± 0.21	2.37–3.07	NS	2.81 ± 0.31	2.30–3.14	L < E, M
C10:0	8.56 ± 0.91	6.79–9.58	NS	9.15 ± 0.74	8.07–9.96	M > L
C10:1	0.20 ± 0.02 ^b^	0.17–0.23	NS	0.27 ± 0.04 ^a^	0.23–0.33	L > E, M
C12:0	3.76 ± 0.63 ^b^	2.78–4.91	NS	5.33 ± 0.40 ^a^	4.51–5.76	NS
C14:0	8.37 ± 0.99 ^b^	6.71–9.66	S > W	11.20 ± 1.07 ^a^	9.47–12.60	L > M > E
iso C15	0.15 ± 0.03 ^b^	0.11–0.19	S > Sp, W	0.20 ± 0.01 ^a^	0.17–0.21	E < M, L
anteiso C15	0.26 ± 0.04 ^b^	0.20–0.32	S > Sp, W	0.40 ± 0.03 ^a^	0.36–0.45	M > E, L
C14:1	0.10 ± 0.01 ^b^	0.08–0.12	NS	0.19 ± 0.08 ^a^	0.11–0.33	L > E, M
C15:0	0.66 ± 0.08 ^b^	0.57–0.77	S > Sp, W	1.09 ± 0.09 ^a^	0.95–1.23	L > M > E
iso C16	0.19 ± 0.01 ^b^	0.17–0.21	S > W	0.23 ± 0.01 ^a^	0.21–0.25	NS
C16:0	25.35 ± 1.51	22.83–28.20	NS	24.08 ± 1.28	22.39–26.24	L > E, M
iso C17	0.33 ± 0.04	0.28–0.39	Sp < S, W	0.33 ± 0.02	0.29–0.36	NS
C16:1	0.52 ± 0.04 ^b^	0.48–0.59	W > Sp, S	0.87 ± 0.16 ^a^	0.71–1.14	L > E, M
anteiso C17	0.35 ± 0.04	0.31–0.43	NS	0.38 ± 0.01	0.35–0.40	NS
C17:0	0.47 ± 0.04 ^b^	0.41–0.53	S > Sp	0.55 ± 0.05 ^a^	0.51–0.67	NS
C17:1	0.19 ± 0.03 ^b^	0.15–0.22	W > Sp	0.23 ± 0.03 ^a^	0.21–0.29	NS
C18:0	10.79 ± 1.05 ^a^	9.93–12.47	W > Sp, S	7.41 ± 0.47 ^b^	6.76–8.20	NS
C18:1 t9	0.25 ± 0.04	0.21–0.30	W > Sp, S	0.22 ± 0.03	0.19–0.30	NS
C18:1 t10	0.45 ± 0.09 ^b^	0.32–0.61	Sp > S	0.71 ± 0.23 ^a^	0.49–1.21	NS
C18:1 t11	1.33 ± 0.22 ^b^	1.07–1.73	W > S	2.03 ± 0.56 ^a^	1.15–2.92	E > L
C18:1 c9	21.64 ± 2.16 ^a^	19.09–25.60	W > Sp, S	15.04 ± 1.09 ^b^	13.89–17.45	NS
C18:1 c11	0.33 ± 0.04 ^b^	0.27–0.41	NS	0.42 ± 0.02 ^a^	0.40–0.44	NS
C18:2 n6	3.61 ± 0.59 ^a^	2.88–4.69	Sp > S, W	1.94 ± 0.24 ^b^	1.67–2.35	E > M, L
C20:0	0.18 ± 0.01 ^a^	0.17–0.20	W > Sp	0.15 ± 0.02 ^b^	0.12–0.18	L > E, M
C18:3 n3	0.42 ± 0.04 ^b^	0.37–0.51	NS	1.06 ± 0.08 ^a^	0.96–1.20	E > M
CLA	0.79 ± 0.10 ^b^	0.67–0.94	W > Sp, S	1.15 ± 0.20 ^a^	0.86–1.47	NS
C20:4 n6	0.21 ± 0.02 ^a^	0.19–0.24	NS	0.16 ± 0.01 ^b^	0.15–0.18	E > M, L
SFA	67.2 ± 2.8 ^b^	62.9–71.1	NS	69.6 ± 1.5 ^a^	66.5–71.1	E > M
MUFA	25.1 ± 2.4 ^a^	22.0–29.3	W > Sp, S	20.0 ± 0.9 ^b^	19.2–22.2	NS
PUFA	5.1 ± 0.6	4.4–6.3	NS	4.7 ± 0.4	4.3–5.3	E > M, L
C10:1/C10:0	0.024 ± 0.001 ^b^	0.022–0.026	NS	0.030 ± 0.006 ^a^	0.025–0.041	L > E, M
C14:1/C14:0	0.012 ± 0.001 ^b^	0.011–0.013	NS	0.016 ± 0.005 ^a^	0.012–0.026	L > E, M
C16:1/C16:0	0.021 ± 0.002 ^b^	0.017–0.024	W > Sp, S	0.036 ± 0.005 ^a^	0.030–0.043	L > E, M
C18:1 c9/C18:0	2.01 ± 0.02	1.92–2.13	NS	2.03 ± 0.15	1.84–2.29	L > M
CLA/C18:1 t11	0.59 ± 0.01	0.54–0.65	S > Sp, W	0.58 ± 0.07	0.51–0.74	L > E
LMF	56.1 ± 3.0 ^b^	53.4–60.4	W > Sp, S	59.4 ± 1.7 ^a^	55.4–61.1	NS
MMF	28.6 ± 2.8	24.6–32.2	W < Sp, S	26.3 ± 2.0	23.6–30.1	NS
HMF	15.3 ± 0.7	14.5–16.6	NS	14.4 ± 1.3	12.7–13.7	L > E, M

^a,b^ Different superscripts indicate significant differences between goat and sheep milks (*p* < 0.05, *t* test); superscript “a” denotes a significantly higher mean, whereas “b” denotes a lower one. * Indicates significant pairwise differences between different seasons using a one-way ANOVA with Tukey’s test (*p* < 0.05). SD, standard deviation; Sp, spring; S, summer; W, winter; E, early season; M, mid-season; L, late season; NS, nonsignificant; CLA, *cis-*9,*trans-*11 conjugated linoleic acid; SFA, saturated fatty acids; MUFA, monounsaturated fatty acids; PUFA, polyunsaturated fatty acids; LMF, low-melting fraction; MMF, medium-melting fraction; HMF, high-melting fraction.

**Table 3:** Protein compositions of goat and sheep milks (indicated by the percentage of HPLC peak area of individual proteins in all identified proteins).

	Goat		Sheep	
	Mean ± SD	Seasonal Effect *	Mean ± SD	Seasonal Effect *
κ-Casein	16.6 ± 0.7	S > Sp > W	11.2 ± 1.0	E < M, L
Serum κ-casein (% of total κ-casein)	22.5 ± 4.9	Sp > S, W	8.1 ± 1.8	NS
α_s1_-Casein	8.9 ± 0.4	NS	34.1 ± 1.1	NS
α_s2_-Casein	10.9 ± 0.4	NS	11.9 ± 1.0	NS
β-Casein	44.2 ± 0.8	NS	29.0 ± 0.5	NS
Serum β-casein (% of total β-casein)	16.0 ± 4.3	NS	6.7 ± 2.3	E > M, L
α-Lactalbumin	8.3 ± 0.4	NS	4.7 ± 0.5	NS
β-Lactoglobulin	11.1 ± 0.8	W > S, Sp	9.1 ± 0.5	L > E, M

* Indicates significant pairwise differences between seasons using a one-way ANOVA with Tukey’s test (*p* < 0.05). SD, standard deviation; Sp, spring; S, summer; W, winter; E, early season; M, mid-season; L, late season; NS, nonsignificant; HPLC, high-performance liquid chromatography.

**Table 4:** Physicochemical properties of goat and sheep milks.

	Goat		Sheep	
	Mean ± SD	Seasonal Effect *	Mean ± SD	Seasonal Effect *
pH	6.67 ± 0.03	NS	6.60 ± 0.05	L < E, M
Fat globule size (µm, D[4,3])	4.00 ± 0.07 ^b^	NS	4.50 ± 0.12 ^a^	E > L
Ethanol stability (%)	48.6 ± 2.5	Sp > S, W	51.4 ± 2.8	L < E, M
Casein micelle size (nm)	212 ± 9 ^a^	NS	179 ± 5 ^b^	E > L > M
Viscosity (mPa⋅s)	3.75 ± 0.12 ^b^	NS	4.64 ± 0.24 ^a^	L > E

^a, b^ Different superscripts indicate significant differences between goat milk and sheep milk (*p* < 0.05, *t* test); superscript “a” denotes a significantly higher mean, whereas “b” denotes a lower one. * Indicates significant pairwise differences between seasons using a one-way ANOVA with Tukey’s test (*p* < 0.05). SD, standard deviation; Sp, spring; S, summer; W, winter; E, early season; M, mid-season; L, late season; NS, nonsignificant; D[4,3], volume-weighted mean diameter.

## Data Availability

Data generated during the study are available from the corresponding author upon request.
